# Role of Neutrophil-to-Lymphocyte Ratio and Platelet-to-Lymphocyte Ratio in Predicting In-Hospital Mortality in Patients With Diabetic Foot Ulcers Undergoing Amputation: A Retrospective Study

**DOI:** 10.7759/cureus.101089

**Published:** 2026-01-08

**Authors:** Bharath Nagarajan, Bhagyasri P, Abhinaya Reddy, Uday Kumbhar, Rajeswari Murugesan

**Affiliations:** 1 Surgery, Jawaharlal Institute of Postgraduate Medical Education and Research, Pondicherry, IND; 2 Biostatistics, All India Institute of Medical Sciences, Mangalagiri, IND

**Keywords:** antimicrobial susceptibility pattern, dfu (diabetic foot ulcer), major limb amputation, predictive risk factors, prognostic biomarkers, wagener’s classification

## Abstract

Background: Diabetic foot ulcers (DFUs) are a severe complication of diabetes and a major cause of non-traumatic lower limb amputations worldwide. Patients undergoing major lower limb amputations (MLLAs) for DFUs often have poor prognoses, with high in-hospital mortality rates. Inflammatory biomarkers, such as the neutrophil-to-lymphocyte ratio (NLR) and platelet-to-lymphocyte ratio (PLR), have emerged as potential prognostic indicators in various clinical settings; however, their predictive value in this high-risk population remains underexplored.

Methodology: This retrospective observational study was conducted at a tertiary care center in South India and included all adult patients with diabetes and DFUs who underwent MLLA between January 2019 and December 2022. Data were extracted from a prospectively maintained database. Postoperative NLR and PLR were calculated from the first postoperative day blood counts. Receiver operating characteristic (ROC) curve analysis determined optimal cut-off values for predicting in-hospital mortality. Variables significant on univariate analysis were entered into a multivariate Cox regression model to identify independent predictors of survival. Microbiological culture and antibiotic sensitivity data from ulcer specimens were also analyzed.

Results: Of the 285 patients (mean age, 55.95 ± 10.42 years), 202 (71%) were male, and 47 (16%) died during hospitalization. ROC analysis revealed that NLR had an area under the curve (AUC) of 0.80, with an optimal cut-off point of 9.1 (sensitivity, 89.4%; specificity, 75.6%). In comparison, PLR had an AUC of 0.686 with a cut-off of 302.8 (sensitivity, 68.1%; specificity, 60.1%). On multivariate Cox regression, recently diagnosed diabetes mellitus (HR = 58.009, p=0.001), high NLR (HR = 12.07; p < 0.001), and high PLR (HR = 2.13; p = 0.012) independently predicted in-hospital mortality. Microbiological analysis yielded a total of 926 organisms isolated from 285 patients. The most common pathogens were *Enterococcus* spp. (21%, n = 194), *Escherichia coli* (18%, n = 170), and *Pseudomonas aeruginosa* (17%, n = 155). *Enterococcus* spp. were most often susceptible to ampicillin, linezolid, and vancomycin.

Conclusion: Elevated postoperative NLR and PLR are independent predictors of in-hospital mortality in DFU patients undergoing MLLA. These readily available biomarkers can assist in early risk stratification and guide postoperative management. The study also provides region-specific data on microbial prevalence and antibiotic susceptibility, supporting more targeted empiric therapy in this high-risk population.

## Introduction

Diabetes mellitus (DM) is one of the most prevalent non-communicable chronic diseases, with a rapidly increasing global burden. According to the International Diabetes Federation 2019 data, approximately 537 million adults worldwide are living with DM, making it the ninth leading cause of mortality [1]. The rising prevalence of DM, coupled with increased life expectancy in affected individuals, has led to a significant increase in diabetic complications, including diabetic foot ulcers (DFUs) [2].

DFUs often progress to severe infections and peripheral vascular disease, accounting for 61.0% to 92.5% of major lower limb amputations (MLLAs) [3]. Alarmingly, it is estimated that a lower limb is amputated due to DM-related complications every 30 seconds worldwide [2]. Postoperative mortality following amputation in diabetic patients ranges from 3.5% to 34%, significantly higher than the <10% mortality rate associated with other types of surgeries [4].

The need for biomarkers to predict mortality following MLLA in diabetic patients is urgent, as early identification of high-risk individuals can guide interventions to reduce mortality and improve postoperative outcomes. Novel biomarkers, such as the neutrophil-to-lymphocyte ratio (NLR) and platelet-to-lymphocyte ratio (PLR), have gained attention as predictors of mortality in conditions like cardiovascular diseases and cancers [5]. These biomarkers are readily accessible through routine blood investigations, yet the precise mechanisms linking elevated NLR and PLR to increased mortality remain unclear.

To date, the relationship between NLR and PLR as predictors of mortality in patients undergoing MLLA has not been widely studied or explored. This study aimed to investigate the role of NLR and PLR in predicting in-hospital mortality in patients undergoing MLLA for DFUs.

## Materials and methods

This retrospective observational study was conducted at a tertiary care center in South India using case records from a prospectively maintained database of patients who underwent MLLA between January 2019 and December 2022. The analysis was carried out between January and May 2024. Approval for the study was obtained from the Institutional Ethics Committee, and a waiver of informed consent was granted due to its retrospective design.

The study population comprised all adult patients above 18 years of age with DM and DFU who underwent MLLA during the study period. MLLA was defined as any amputation performed at or proximal to the ankle joint. Patients who underwent amputation for causes other than diabetic foot disease, such as trauma or malignancy, were excluded. Patients with incomplete or non-retrievable medical records were also excluded.

Data were retrieved from patient case records and included demographic and clinical information such as age, gender, anthropometric parameters, type and duration of DM, history of previous amputations, alcohol intake, and smoking status. DFUs were classified according to Wagner’s grading system, which assesses the depth of the ulcer and the presence or absence of osteomyelitis [6]. Laboratory investigations included blood urea, serum creatinine, albumin, direct bilirubin, and indirect bilirubin levels.

Postoperative inflammatory markers were assessed using laboratory values obtained on the first postoperative day. The NLR was calculated as the ratio of absolute neutrophil count to absolute lymphocyte count, while the PLR was calculated as the ratio of platelet count to absolute lymphocyte count.

Hospital outcomes were recorded as discharge or in-hospital death, whichever occurred first. Additionally, microbiological data were obtained from DFU cultures, including the isolated organisms and their corresponding antibiotic sensitivity patterns.

Statistical analysis was performed using IBM SPSS Statistics for Windows, version 29 (IBM Corp., Armonk, NY, USA). Continuous variables were expressed as mean ± standard deviation or median with interquartile range, depending on data distribution. Cut-off values for the NLR and PLR were determined using receiver operating characteristic (ROC) curve analysis, and patients were categorized into high and low NLR/PLR groups. Univariate Cox proportional hazards regression was performed to identify factors associated with in-hospital mortality. Variables with a p-value < 0.10 on univariate analysis were then entered into a multivariate Cox proportional hazards regression model. A two-tailed p-value < 0.05 was considered statistically significant.

## Results

During the study period, a total of 285 patients underwent MLLAs for diabetic foot infections (DFIs) and were included in the analysis. The mean age of the cohort was 55.95 ± 10.42 years, with a male predominance (n = 202, 71%). Among these patients, 269 (94%) had a prior diagnosis of DM, while 16 (6%) were newly diagnosed following the development of foot infections. Most patients (n = 276, 96%) had a history of previous hospital admission for DFIs, and 271 (95%) had undergone either debridement or minor amputations before requiring MLLA. Below-knee amputations were the most common (n = 236, 83%), while the remainder were above-knee amputations. The detailed demographic and clinical characteristics of the study population are presented in Table 1.

**Table 1:** Demographic and clinical characteristics of the study population. SD, standard deviation.

Variable	Category	Value, n (%)
Age (years)	Mean ± SD	55.95 ± 10.40
Gender	Male	202 (70.9)
	Female	83 (29.1)
Diabetic status	Known case	269 (94.4)
	Newly diagnosed	16 (5.6)
Type of diabetes mellitus	Type 1	1 (0.4)
	Type 2	284 (99.6)
Treatment among known diabetics (n = 269)	No treatment	21 (7.8)
	Oral hypoglycemic agents	200 (74.3)
	Insulin	48 (17.8)
Smoking status	No	251 (88.1)
	Yes	34 (11.9)
Hypertension	No	187 (65.6)
	Yes	98 (34.4)
Coronary artery disease	No	254 (89.1)
	Yes	31 (10.9)
Cerebrovascular accident	No	279 (97.9)
	Yes	6 (2.1)
Chronic kidney disease	No	266 (93.3)
	Yes	19 (6.7)
Chronic obstructive pulmonary disease	No	283 (99.3)
	Yes	2 (0.7)
Admission for diabetic foot	No	9 (3.2)
	Yes	276 (96.8)
Affected limb	Right	117 (41.1)
	Left	168 (58.9)
Prior debridement/minor amputation	No	14 (4.9)
	Yes	271 (95.1)
Level of amputation	Below knee	236 (82.8)
	Above knee	49 (17.2)
Discharge/death	Death	47 (16.5)
	Discharge	238 (83.5)

**Figure 1:**
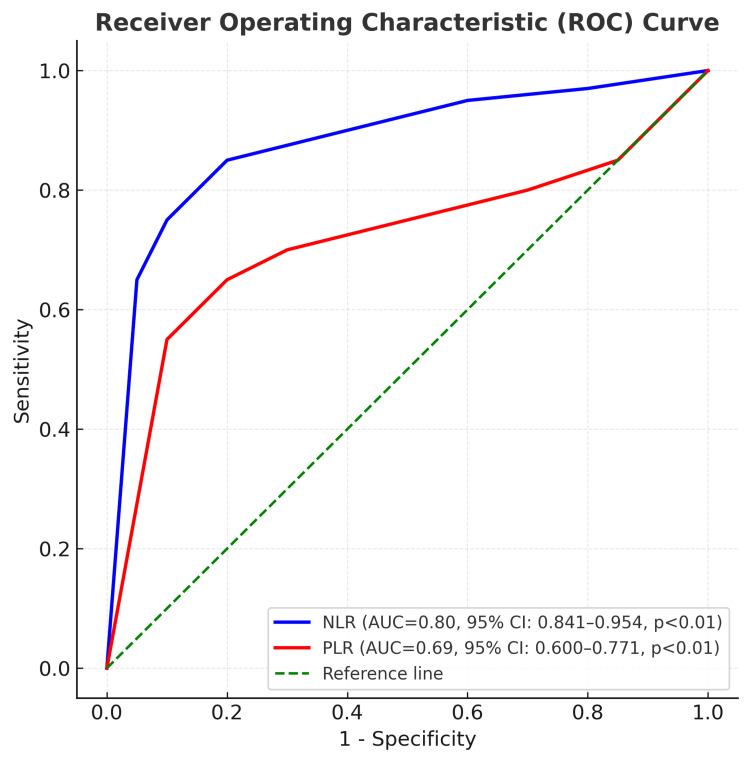
Receiver operating characteristic curves predicting the mortality for baseline NLR and PLR. AUC, area under the curve; NLR, neutrophil-to-lymphocyte ratio; PLR, platelet-to-lymphocyte ratio.

In-hospital mortality occurred in 47 patients (16%) following MLLA. ROC curve analysis demonstrated the predictive ability of the NLR and PLR for mortality. NLR showed an area under the curve (AUC) of 0.80 (95% CI, 0.841-0.954; p < 0.01), with a cut-off value of 9.1 providing 89.4% sensitivity and 75.6% specificity. PLR showed an AUC of 0.686 (95% CI, 0.600-0.771; p < 0.01), with a cut-off value of 302.80 yielding 68.1% sensitivity and 60.1% specificity (Figure 1).

On univariate Cox proportional hazards regression analysis, recently diagnosed DM, smoking, high NLR, high PLR, renal impairment, and liver dysfunction were associated with increased mortality. Variables with a p-value < 0.10 on univariate analysis were entered into the multivariate Cox proportional hazards regression model. On multivariate analysis, recently diagnosed DM (HR 58.009, 95% CI 4.833 -689.103; p=0.001), high NLR (HR 12.07, 95% CI 4.44-32.85; p < 0.001), and high PLR (HR 2.13, 95% CI 1.18-3.86; p = 0.012) remained independently and statistically significant predictors of mortality. Smoking, renal impairment, and liver dysfunction did not retain statistical significance after adjustment. The complete results of the univariate and multivariate analyses are presented in Table 2.

**Table 2:** Univariate and multivariate analyses of factors affecting the in-hospital mortality in study population. NLR, neutrophil-to-lymphocyte ratio; PLR, platelet-to-lymphocyte ratio; CI, confidence interval. Renal impairment: Acute or chronic elevation in blood urea nitrogen (BUN) and/or serum creatinine from baseline (BUN >20 mg/dl, serum creatinine > 1.2 mg/dl). Liver dysfunction: Increase in total/direct bilirubin from baseline (total bilirubin : >1.2 mg/dl, direct bilirubin > 0.18 mg/dl). Univariate analysis: Each covariate was initially assessed using univariate Cox proportional hazards regression. Variables were entered individually, and hazard ratios (HRs) with 95% confidence intervals (CIs) and P values were obtained using the Wald test. Multivariate analysis: Variables with P < 0.10 on univariate analysis were included in a multivariate Cox proportional hazards regression model. Adjusted HRs with 95% CIs were estimated using the Wald test. * A p-value < 0.05 was considered statistically significant. NLR and PLR categorization: NLR ≥ 9.1 was defined as high NLR, and NLR < 9.1 as low NLR. PLR ≥ 302.8 was defined as high PLR, and PLR < 302.8 as low PLR.

Covariate	Category	Univariate Cox proportional hazards regression analysis				Multivariate Cox proportional hazards regression analysis			
		Hazard ratio	95% CI		p-value	Hazard ratio	95% CI		p-value*
			Lower	Upper			Lower	Upper	
Age	≤60 years	Reference	-	-	-	-	-	-	-
	>60 years	0.822	0.448	1.509	0.527	-	-	-	-
Sex	Male	1.482	0.768	2.862	0.241	-	-	-	-
	Female	Reference	-	-	-	-	-	-	-
Diabetes mellitus	Recently diagnosed	11.600	1.407	95.609	0.023	58.009	4.883	689.103	0.001
	Known case	Reference	-	-	-	-	-	-	-
If known case	No treatment	Reference	-	-	-	-	-	-	-
	Oral hypoglycemics	0.644	0.154	2.694	0.547	-	-	-	-
	Insulin	0.582	0.125	2.714	0.491	-	-	-	-
Smoker	Yes	2.563	1.293	5.080	0.007	1.626	0.787	3.363	0.189
	No	Reference	-	-	-	-	-	-	-
Hypertension	Yes	1.669	0.935	2.978	0.12	-	-	-	-
	No	Reference	-	-	-	-	-	-	-
Coronary artery disease	Yes	1.182	0.499	2.801	0.704	-	-	-	-
	No	Reference	-	-	-	-	-	-	-
Cerebrovascular accident	Yes	2.117	0.509	8.801	0.302	-	-	-	-
	No	Reference	-	-	-	-	-	-	-
Chronic kidney disease	Yes	1.917	0.854	4.303	0.115	-	-	-	-
	No	Reference	-	-	-	-	-	-	-
Chronic obstructive pulmonary disease	Yes	2.981	0.406	21.897	0.283	-	-	-	-
	No	Reference	-	-	-	-	-	-	-
Admission for diabetic foot	Yes	1.500	0.205	10.971	0.689	-	-	-	-
	No	Reference	-	-	-	-	-	-	-
Debridement	Yes	2.011	0.276	14.670	0.491	-	-	-	-
	No	Reference	-	-	-	-	-	-	-
Previous amputation	Yes	1.771	0.243	12.925	0.573	-	-	-	-
	No	Reference	-	-	-	-	-	-	-
Infection	Present	1.422	0.441	4.588	0.556	-	-	-	-
	Absent	Reference	-	-	-	-	-	-	-
NLR category	High	12.621	4.986	31.949	0.001	12.072	4.437	32.849	<0.001
	Low	Reference	-	-	-	-	-		
PLR category	High	3.054	1.711	5.450	0.001	2.13	1.177	3.856	0.012
	Low	Reference	-	-	-	-	-	-	-
Hemoglobin	≤10 g/dl	1.824	0.442	7.538	0.406	-	-	-	-
	>10 g/dl	Reference	-	-	-	-	-	-	-
Renal impairment		0.994	0.990	0.998	0.006	1	0.993	1.006	0.914
Liver dysfunction	No	Reference	-	-	-	-	-	-	-
	Yes	2.497	1.192	5.230	0.015	1.814	0.836	3.934	0.132
Level of amputation	Below knee	0.597	0.300	1.188	0.142	-	-	-	-
	Above knee	Reference	-	-	-	-	-	-	-
Duration (weeks) since diagnosis of diabetic foot		1.002	0.998	1.006	0.308	-	-	-	-

Microbiological analysis yielded a total of 926 organisms isolated from the 285 patients. Gram-negative organisms accounted for 75% of isolates, while 25% were gram-positive. The most common pathogen was *Enterococcus* spp. (21%), a gram-positive coccus, followed by *Escherichia coli* (18%) and *Pseudomonas aeruginosa* (17%). The detailed microbiological distribution is presented in Table 3.

**Table 3:** Pathogens isolated from wound cultures of the study population.

Bacteria	Value, n (%)
Gram-positive organisms	
Enterococcus spp.	194 (21.0)
Staphylococcus aureus	18 (1.9)
Methicillin-resistant Staphylococcus aureus (MRSA)	19 (2.1)
Gram-negative organisms	
Escherichia coli	170 (18.4)
Klebsiella pneumoniae	95 (10.3)
Pseudomonas aeruginosa	155 (16.7)
Acinetobacter baumannii	100 (10.8)
Proteus spp.	114 (12.3)
Morganella morganii	33 (3.6)
Providencia spp.	28 (3.0)
Total	926 (100)

Antibiotic sensitivity testing revealed varied patterns of resistance. Among the gram-positive organisms, *Enterococcus* spp. showed sensitivity to ampicillin (29%), linezolid (23%), and vancomycin (23%). *Staphylococcus aureus* and methicillin-resistant *Staphylococcus aureus* (MRSA) demonstrated heterogeneous sensitivity to clindamycin, cotrimoxazole, and erythromycin, as detailed in Table 4.

**Table 4:** Antibiotic sensitivity pattern of gram-positive organisms of the study population. MRSA, methicillin-resistant Staphylococcus aureus.

Agent	Staphylococcus aureus (n = 18), n (%)	MRSA (n = 19), n (%)	Enterococcus spp. (n = 194), n (%)
Ampicillin	0 (0)	0 (0)	57 (29.4)
Linezolid	3 (16.7)	5 (26.3)	44 (22.7)
Vancomycin	0 (0)	5 (26.3)	45 (23.2)
Teicoplanin	0 (0)	0 (0)	41 (21.1)
Amikacin	0 (0)	0 (0)	3 (1.5)
Cefoperazone–sulbactam	0 (0)	0 (0)	1 (0.5)
Ceftazidime	0 (0)	0 (0)	1 (0.5)
Cefoperazone	0 (0)	0 (0)	0 (0)
Piperacillin–tazobactam	0 (0)	0 (0)	1 (0.5)
Meropenem	0 (0)	0 (0)	0 (0)
Minocycline	0 (0)	0 (0)	1 (0.5)
Clindamycin	4 (22.2)	2 (10.5)	–
Cotrimoxazole	5 (27.8)	3 (15.8)	–
Erythromycin	4 (22.2)	4 (21.1)	–
Oxacillin	2 (11.1)	0 (0)	–

Among the gram-negative organisms, *Escherichia coli* demonstrated the highest sensitivity to amikacin (34%), followed by cefoperazone-sulbactam (19%) and piperacillin-tazobactam (16%). *Acinetobacter baumannii* showed predominant sensitivity to minocycline (70%) and to a lesser extent to amikacin (10%). The complete antibiotic sensitivity patterns for gram-negative organisms are summarized in Table 5.

**Table 5:** Antibiotic sensitivity pattern of gram-negative organisms of the study population.

Agent	Escherichia coli (n = 170), n (%)	Klebsiella pneumoniae (n = 95), n (%)	Pseudomonas aeruginosa (n = 155), n (%)	Acinetobacter baumannii (n = 100), n (%)	Proteus spp. (n = 114), n (%)	Morganella morganii (n = 33), n (%)	Providencia spp. (n = 28), n (%)
Ampicillin	4 (2.4)	1 (1.1)	0 (0)	0 (0)	0 (0)	1 (3.0)	0 (0)
Linezolid	3 (1.8)	1 (1.1)	0 (0)	0 (0)	1 (0.9)	0 (0)	0 (0)
Vancomycin	3 (1.8)	1 (1.1)	0 (0)	0 (0)	0 (0)	0 (0)	0 (0)
Teicoplanin	1 (0.6)	1 (1.1)	0 (0)	0 (0)	0 (0)	0 (0)	0 (0)
Amikacin	57 (33.5)	35 (36.8)	41 (26.5)	10 (10.0)	24 (21.1)	11 (33.3)	5 (17.9)
Cefoperazone–sulbactam	32 (18.8)	17 (17.9)	27 (17.4)	4 (4.0)	18 (15.8)	4 (12.1)	4 (14.3)
Ceftazidime	8 (4.7)	10 (10.5)	32 (20.6)	5 (5.0)	23 (20.2)	5 (15.2)	3 (10.7)
Cefoperazone	12 (7.1)	9 (9.5)	7 (4.5)	4 (4.0)	6 (5.3)	4 (12.1)	1 (3.6)
Piperacillin–tazobactam	28 (16.5)	15 (15.8)	38 (24.5)	3 (3.0)	27 (23.7)	5 (15.2)	7 (25.0)
Meropenem	21 (12.4)	5 (5.3)	10 (6.5)	4 (4.0)	14 (12.3)	3 (9.1)	8 (28.6)
Minocycline	1 (0.6)	0 (0)	0 (0)	70 (70.0)	1 (0.9)	0 (0)	0 (0)

## Discussion

This retrospective observational study assessed the predictive value of postoperative NLR and PLR for in-hospital mortality among patients undergoing MLLAs for DFIs. The results demonstrate that NLR values above 9.1, PLR values above 302.80, and recently diagnosed DM serve as independent risk factors for mortality in this population. Although chronic kidney disease, hypertension, and smoking history were associated with increased mortality on univariate analysis, they were not found to be statistically significant in multivariate models.

NLR and PLR are emerging inflammatory markers that have been investigated in cardiovascular diseases, cancers, and sepsis [5], but their application in DFU populations remains limited. The biological plausibility for their predictive role lies in the systemic inflammatory and immune dysregulation caused by hyperglycemia. Poorly controlled diabetes results in persistent inflammation, abnormal neutrophil activation, endothelial dysfunction, and oxidative stress, which manifest as neutrophilia, platelet activation, and lymphocyte depletion through apoptosis. These mechanisms collectively contribute to elevated NLR and PLR values [7].

Several prior studies have evaluated the prognostic significance of NLR in DFIs and amputations. Tasoglu et al. and Demirdal & Sen reported NLR as a predictor of amputation risk in patients with critical limb ischemia and acute arterial occlusion [8,9]. Yapoci et al. [10] found NLR to be independently associated with osteomyelitis and progression to amputation in DFIs, while Aircan et al. [7] identified a cut-off of 6.73 as a predictor for major amputations in DFIs. Dinc et al. reported NLR as an independent predictor of mortality following non-traumatic major lower extremity amputation, with a cut-off value of 6.8 [11]. In contrast, fewer studies have examined PLR. Wang et al. and Hudzik et al. highlighted the role of PLR in predicting mortality in sepsis and diabetes, respectively [12,13]. The present study builds upon this evidence by explicitly addressing the predictive utility of both NLR and PLR in the postoperative outcomes of patients undergoing MLLA for DFIs.

When compared with prior literature, this study is among the few to evaluate NLR and PLR in relation to MLLA outcomes. Chen et al. reported lower cut-off values for NLR (2.76) and PLR (160.05), although with reduced sensitivity and specificity compared with the present findings [14]. Variations in patient populations, severity of infection, and methodological differences likely account for these discrepancies. Consistent with Chen et al., however, this study demonstrated a strong association between elevated NLR, advanced Wagner classification, and mortality, underscoring the robustness of NLR as a prognostic marker [14].

The microbiological profile in this study revealed a predominance of gram-negative organisms (75%), while gram-positive organisms accounted for 25%. *Enterococcus* spp. emerged as the most frequent isolate (21%), followed by *Escherichia coli* (18%) and *Pseudomonas aeruginosa* (17%). These findings contrast with earlier studies that identified *Staphylococcus aureus* as the predominant organism [15]. The difference likely reflects the advanced disease stage and higher Wagner grades observed in the present cohort. Notably, the antibiotic sensitivity patterns indicated worrisome levels of resistance. Only 21% of *Enterococcus* spp. isolates were sensitive to vancomycin, while *Acinetobacter baumannii* exhibited high resistance to most empirical agents, with only 10% sensitivity to amikacin and 70% sensitivity to minocycline. These observations highlight the urgent need for region-specific antibiograms to inform empiric therapy in patients with DFIs.

From a clinical perspective, these findings have two key implications. First, NLR and PLR can serve as simple, cost-effective, and readily available biomarkers to identify high-risk patients who require closer monitoring and more aggressive management strategies. Their ease of calculation from routine blood investigations makes them particularly attractive for use in resource-limited settings. Second, the antibiotic resistance trends emphasise the importance of antimicrobial stewardship and the development of local antibiograms to guide empirical therapy and reduce mortality associated with inappropriate antibiotic use.

The study has certain limitations. Its retrospective design precludes assessment of dynamic changes in NLR and PLR over the course of hospitalization. Being a single-center study, the findings may not be generalizable to other populations or healthcare settings. Additionally, some important clinical variables, such as neuropathy and microangiopathy, which significantly influence the outcomes of DFIs, were not analyzed.

Future research should focus on prospective multicenter studies with larger sample sizes to validate these results and evaluate the temporal changes in NLR and PLR during hospitalization. Incorporating additional biomarkers, imaging modalities, and clinical risk factors into predictive models could further refine prognostication and improve management strategies for patients with DFIs undergoing MLLA.

## Conclusions

This retrospective study highlights the utility of NLR and PLR as valuable prognostic markers in patients with DFIs and MLLAs. Elevated NLR and PLR were shown to independently predict in-hospital mortality, underlining their potential clinical relevance in risk stratification for this high-risk patient population. To further validate these findings and enhance their applicability, prospective, large-scale studies are needed. Such studies would establish more robust predictive models and refine the optimal cut-off values for NLR and PLR, enabling more precise identification of at-risk individuals. By leveraging these biomarkers, clinicians can implement targeted interventions, provide vigilant monitoring, and tailor management strategies to improve patient outcomes and reduce complications associated with DFIs and MLLAs.
